# Generation and Characterization of a CRISPR/Cas9—Induced *3-mst* Deficient Zebrafish

**DOI:** 10.3390/biom10020317

**Published:** 2020-02-17

**Authors:** Antonia Katsouda, Maria Peleli, Antonia Asimakopoulou, Andreas Papapetropoulos, Dimitris Beis

**Affiliations:** 1 Center for Clinical, Experimental Surgery & Translational Research, Biomedical Research Foundation, Academy of Athens, 11527 Athens, Greece; antonia.katsouda@gmail.com (A.K.); mpeleli@bioacademy.gr (M.P.); 2 Laboratory of Pharmacology, Faculty of Pharmacy, National and Kapodistrian University of Athens, 15771 Athens, Greece; antonia.asim@gmail.com

**Keywords:** 3-mercaptopyruvate sulfurtransferase, hydrogen sulfide, reactive oxygen species, zebrafish

## Abstract

3-mercaptopyruvate sulfurtransferase (3-MST) is an enzyme capable of synthesizing hydrogen sulfide (H_2_S) and polysulfides. In spite of its ubiquitous presence in mammalian cells, very few studies have investigated its contribution to homeostasis and disease development, thus the role of 3-MST remains largely unexplored. Here, we present a clustered, regularly interspaced, short palindromic repeats (CRISPR)/CRISPR–associated protein-9 (Cas9) induced *3-mst* mutant zebrafish line, which will allow the study of 3-MST’s role in several biological processes. The *3-mst* zebrafish orthologue was identified using a bioinformatic approach and verified by its ability to produce H_2_S in the presence of 3-mercaptopyruvate (3-MP). Its expression pattern was analyzed during zebrafish early development, indicating predominantly an expression in the heart and central nervous system. As expected, no detectable levels of 3-Mst protein were observed in homozygous mutant larvae. In line with this, H_2_S levels were reduced in *3-mst^−/−^* zebrafish. Although the mutants showed no obvious morphological deficiencies, they exhibited increased lethality under oxidative stress conditions. The elevated levels of reactive oxygen species, detected following *3-mst* deletion, are likely to drive this phenotype. In line with the increased ROS, we observed accelerated fin regenerative capacity in *3-mst* deficient zebrafish. Overall, we provide evidence for the expression of *3-mst* in zebrafish, confirm its important role in redox homeostasis and indicate the enzyme’s possible involvement in the regeneration processes.

## 1. Introduction

After the discovery that H_2_S is endogenously produced in mammalian cells, the number of studies investigating its biological actions has been steadily increasing, providing new information on its role in a wide range of physiological and pathological states [1]. H_2_S is now recognized as a biological mediator, which interacts with numerous molecular targets and impacts on most organ systems; together with nitric oxide and carbon monoxide, it is part of the gasotrasmitter family [2,3]. Cystathionine-gamma-lyase (CSE), cystathinine-beta-synthetase (CBS) and 3-mercaptopyruvate sulfutransferase (3-MST) are the principal enzymes that contribute to the endogenous H_2_S production [4,5]. CBS and CSE participate in several H_2_S-generating reactions using cysteine and/or homocysteine as substrates, while 3-MST catalyzes the production of H_2_S, polysulfides and persulfides via 3-MP utilization [5]. The three enzymes are differentially regulated and expressed at different levels in each tissue [5,6]. It is important to note that CSE, CBS and 3-MST differ in their subcellular localization. Under resting conditions, CSE and CBS reside in the cytosol, while 3-MST is also present in the mitochondria [7]. Although the role of CBS and CSE has been extensively studied, very little is known about the function and signaling of 3-ΜST. For many years, the lack of 3-MST genetic animal models and specific pharmacological inhibitors/activators has slowed down progress in the field. Recently, a 3-MST knockout mouse was generated and compounds that selectively inhibit 3-MST have been reported [8,9]. These tools are expected to significantly contribute to the clarification of the biological role of 3-MST.

Zebrafish is a popular vertebrate model system with a high physiological and genetic similarity to mammals [10]. It provides multiple advantages in modeling human disease, including external fertilization, a large number of offspring, rapid rate of development and transparent embryos that allow for non-invasive in vivo imaging [11,12]. Furthermore, zebrafish is not dependent on a fully functional cardiovascular system for the first days of its development and maintains the ability to regenerate most of its organs throughout its lifetime [12,13,14,15]. Thus, it facilitates the study of biological processes that could not be investigated in mammalian models. Importantly, the easy genetic manipulation of zebrafish via forward and reverse genetic approaches has significantly contributed to further expanding the knowledge of various molecular pathways [16,17,18,19]. In the present study, we generated and initiated the characterization of a clustered, regularly interspaced, short palindromic repeats (CRISPR)/Cas9 engineered *3-mst* loss-of-function mutant zebrafish, a new model that will allow a deeper understanding of 3-MST’s function.

## 2. Materials and Methods

### 2.1. Zebrafish Maintenance and Breeding 

Zebrafish embryos were raised under standard laboratory conditions at 28 °C and maintained in accordance with the European Directive 2010/63 for the protection of animals used for scientific purposes and the Recommended Guidelines for Zebrafish Husbandry Conditions [20]. The genetic background used was wild-type Ab strain, and the allele generated and described here is assigned as *3-mst* (aa102). The fish were raised in the animal facility of BRFAA and the zebrafish experimental protocols were approved by the BRFAA ethics committee and the Attica Veterinary Department (EL25BIO003). The adult regeneration experiments were approved on 24-10-2018 (no.5519). The chemical treatment experimentations described in this study were completed by day five of the zebrafish embryo development. Therefore, these experiments are not considered animal experiments and do not fall under the protection guidelines of the directive 2010/63/EU, revising directive 86/609/EEC, on the protection of animals used for scientific purposes as adopted on 22 September 2010.

### 2.2. Protein Expression and Purification 

The expression and purification of *zgc162544* were performed as described previously with some modifications [21]. Briefly, E. coli BL21 (DE3) Codon Plus (StrataGene, USA) was used as the host strain to express recombinant Zgc1625144. *Zgc162544* cDNA (Scource Bioscience) was cloned into pGEX-Kg to create N-terminal GSH-S-transferase (GST) fusion proteins. The expression vectors were transformed and plated on Luria–Bertani (LB)-agar plates, supplemented with 100 mg/mL ampicillin (Applichem Biochemica). Codon Plus cells containing pGEX-Kg/GST-Zgc162544 were grown at 37 °C and 180 r.p.m. in LB broth medium containing 100 μg/mL ampicillin to an absorption of 0.6–0.8 at 600 nm. The protein expression was induced by the addition of 0.1 mM Isopropyl-b-d-thiogalactopyranoside (IPTG) (Applichem Biochemica) and cells were incubated overnight at 20 °C. The overnight culture was then centrifuged at 4 °C and 8000× *g* for 15 min and the cell pellet was resuspended in Phosphate Buffered Saline, PBS (140 mM NaCl, 2.7 mM KCl, 10 mM Na_2_HPO4, 1.8 mM KH_2_PO4, pH 7.8) containing protease inhibitors cocktail (Sigma-Aldrich) and sonicated. After centrifugation at 4 °C for 30 min, the soluble fraction was filtered (0.2 μΜ) and loaded onto a GSTrap FF 1 mL affinity column (GE Healthcare, Sweden), previously equilibrated with binding buffer PBS. The column was consecutively washed with five column volumes of binding buffer. Proteins attached to the column, including GST-Zgc162544 recombinant protein, were eluted with five column volumes of elution buffer (50 mM Tris–HCl, 10 mM reduced GSH (Sigma-Aldrich), pH 8.0) and then dialysed and concentrated in 10 mM sodium phosphate buffer, pH 8.2 and 1 mM DTT (Applichem Biochemica). The purity of the recombinant enzymes was checked by SDS-PAGE on 12% polyacrylamide gels after staining of the protein bands with Coomassie Blue R-250 (Sigma-Aldrich). Protein concentration was determined using the DC protein assay kit (Biorad).

### 2.3. In Situ Hybridization 

Whole-mount in situ hybridization experiments with a *3-mst* antisense probe were performed in different stages in the embryos according to the Thisse protocol for ISH [22].

### 2.4. CRISPR/Cas9 Genome Editing Technique

The CRISPR/Cas9 targeted mutatagenesis was performed according to [23]. Shortly, a guide RNA targeting exon 1 of *3-mst* was designed (ZiFiT-Targeter 4.2) and cloned into a T7-driven promoter expression vector pT7-gRNA (Addgene). The synthesis of mRNA was performed using the T7 RNA polymerase (Roche). Cas9 mRNA was transcribed in vitro from the pT3TS-nCas9n vector (Addgene). For mutant generation, 4.6 nL of a mixture containing 100 ng/μL guide RNA and 150 ng/μL Cas9-mRNA was injected into the cell of οne-cell stage embryos. Oligonucleotides sequences: gRNA F: 5′ TAGGGCGAGTTTGCAGACTATG 3, gRNA R: 5′ AAACCATAGTCTGCAAACTCGC 3′.

### 2.5. T7 Endonuclease I Assay (T7E1)

A short stretch of genomic region flanking the CRISPR target site was PCR amplified from the genomic DNA, isolated from zebrafish embryos. Purified PCR amplicons were denatured and slowly reannealed to facilitate heteroduplex formation (5 min denaturing step at 95 °C, followed by cooling to 85 °C at −2 °C/sec and further to 25 °C at 0.1 °C/sec). The reannealed amplicon was then digested with 10 units of T7 endonuclease I (New England Biolabs) at 37 °C for 90 min. Results were evaluated by electrophoresis on a 2.5% agarose gel. Primers sequence: *3-MST* F: 5′ CTAACCCTCTGTGTCGGTGT 3′, *3-MST* R: 5′ CACGGTGGCTTCAAGAACTG 3′.

### 2.6. Genotyping

Genotyping was performed using the sequencing analysis (Macrogen) of PCR-products, amplified by oligos which are specific to the respective region of the gene (same as in Section 2.5). Genomic DNA, which is used as a template for PCR, was isolated from adult zebrafish fins. For the fin-clipping procedure, the zebrafish were anesthetized in 0.02% tricaine methanesulfonate (Sigma-Aldrich).

### 2.7. Western Blot Analysis

Zebrafish embryos at 96 h post-fertilization (hpf) were homogenized in lysis buffer (50 mM Tris-HCL pH = 7.4, 1% NP-40, 0.5% Na-deoxycholate, 0.1% SDS, 150 mM NaCL, 2 mM EDTA) supplemented with 1% protease inhibitors cocktail (Sigma-Aldrich). The samples were separated on 10% SDS-PAGE and transferred to a nitrocellulose membrane (Macherey-Nagel). The membrane was blocked and probed with the following antibodies at the indicated dilutions: anti-3-MST (1:500, Atlas Antibodies, Product Number: HPA001240) and β-tubulin (1:1000, Abcam, Product Number: ab15568). The immunoblots were next processed with a secondary antibody (1:4000, Millipore, Product Number: AP132P) and visualized using Western HRP substrate (Millipore).

### 2.8. Measurement of H_2_S Production Using the Methylene Blue Assay

H_2_S determination was performed as we previously described with slight modifications [21,24]. In brief, zebrafish embryos at 96 hpf were collected and homogenized in ice-cold 100 mM potassium phosphate buffer, pH = 7.4. Samples were prepared in parafilm-sealed eppendorf tubes containing embryos’ homogenates, 8μM pyridoxal-5′-phosphate, 0.4 mM l-cysteine, 0.08 mM homocysteine and 8 μM 3-mercaptopyruvate. After 30 min of incubation at 37 °C in a shaking water bath, the reaction was terminated by adding 1% zinc acetate to trap H_2_S, followed by 10% trichloroacetic acid to precipitate proteins. Subsequently, *N,N*-dimethyl-*p*-phenylenediamine-sulfate in 7.2 M HCl was added, followed by FeCl_3_ in 1.2 M HCl. Absorbance was measured at 655 nm and H_2_S content was calculated against a calibration curve of standard H_2_S solutions. Results were expressed as concentration of H_2_S formed per μg protein. Protein concentration was determined using a Bradford Assay. In the case of the GST-Zgc162544 enzyme, each test, containing 5μg of the purified enzyme, 15 μM 3-MP and 50 mM sodium phosphate buffer pH 8.2, was incubated at 28 °C for 1 h. GST was not removed from the fusion proteins as it has been previously proven that the presence of GST does interfere with the assay, or affects enzymes’ activity [25]. All reagents for the methylene blue assay were from Sigma-Aldrich.

### 2.9. Measurement of H_2_S Production Using the AzMC Probe

Zebrafish embryos at 96 hpf were collected and lysed in ice-cold NP40 lysing buffer (1% NP40; 150 mM NaCl; 50 mM Tris-Cl, pH 8.0). Homogenates were added to the reaction mixture (50 mM Tris HCL pH 8.0), which was supplemented with 2 mM homocysteine, 2 mM l-cysteine, 2 mM 3-mercaptopyruvate and 5 μΜ pyridoxal 5′-phosphate. After 30 min of incubation at 37 °C, AzMc (7-azido-4-methylcoumarin; 100 μM final concentration) was added to samples. Fluorescence was measured under the following settings: ex = 365 nm, em = 450 nm, gain = 60 nm. The results were expressed as concentrations of H_2_S formed per μg protein. Protein concentration was determined using an absorbance of 280 nm. All reagents for this assay were from Sigma-Aldrich.

### 2.10. Paraquat (PQ) Treatment of Zebrafish Embryos

Paraquat (Sigma-Aldrich) was diluted in embryo water to a final concentration of 500 μg/mL. Embryos were treated at 96 hpf for 48 h. Embryo water was used as a control.

### 2.11. Menadione (MN) Treatment of Zebrafish Embryos

Menadione (Sigma-Aldrich) was diluted in dimethyl sulfoxide (DMSO, Sigma-Aldrich). Embryos aged 48 hpf were exposed to 10 μΜ MN (0.01% DMSO in embryo water) for 24 h. For the control, 0.01% DMSO in embryo water was used.

### 2.12. Measurement of Zebrafish Hydrogen Peroxide

The H_2_O_2_ levels measurement was performed as previously described, with some modifications [26]. In particular, zebrafish embryos at 96 hpf were homogenized in PBS, supplemented with protease and phosphatase inhibitors (Sigma-Aldrich) and next centrifuged at 4 °C and 2000× *g* for 10 min. Supernatants were collected and the fluorescence was measured at 37 °C after the addition of 100 μΜ NADPH, 100 μM AmpliFlu Red and 1 U/mL Horse Radish Peroxidase. All reagents were from Sigma-Aldrich. The final RFU signal was normalized according to the protein concentration, which was determined using the Bradford method. The fluorescence was measured over a total time of 60 min under the following settings: ex = 535 nm, em = 595 nm, gain = 50.

### 2.13. Zebrafish Fin Excision

Fin regeneration experiments were performed on adult zebrafish. The fish were anaesthetized in 0.02% tricaine methanesulfonate (Sigma-Aldrich) and their caudal fins were amputated using a scalpel. Animals were allowed to regenerate at 28 °C for 18 days. The efficiency of regeneration was quantified by the measurement of fins’ length at different time points.

### 2.14. Statistical Analysis

Data are presented as mean ± SEM. Differences were analyzed using two-tailed Student’s *t*-test. *p* was considered significant when less than 0.05.

## 3. Results

### 3.1. Identification of the 3-MST Orthologue in Zebrafish

**Figure 1:**
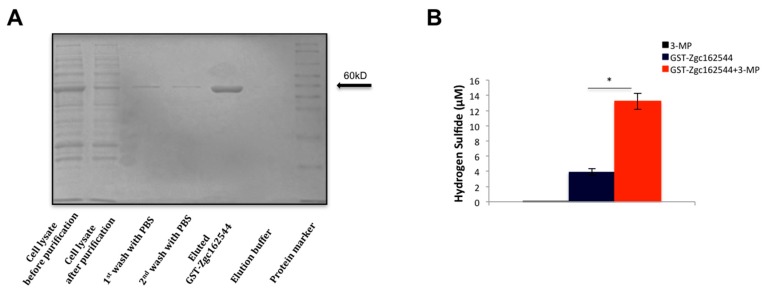
Purification and enzymatic activity of recombinant GST-Zgc162544. (**A**) Representative SDS-PAGE of all fractions collected after different purification steps of GST-Zgc162544. (**B**) H_2_S production by GST-Zgc162544 in the presence of 3-MP. Data are presented as mean ± SEM; *n* = 6–7; * *P* < 0.05.

Two orthologues of the human *CBS* gene (*cbsa* and *cbsb*) and two orthologues of the human *CSE* (the *cth* and *cthl*) exist in the zebrafish genome. These genes are registered in many databases and have been examined in several studies. However, the orthologue of the 3-MST gene in zebrafish has not been identified, yet. In order to identify it, we first used bioinformatic approaches that revealed the *zgc162544* as the strongest candidate gene. *Zgc162544* is 58% homologous at the protein level and has a 77% amino acid identity to human 3-MST (Supplementary Figure S1A). Moreover, it contains two protein thiosulfate sulfurtransferase domains, an inactive and a catalytic one, both of them containing two copies of the rhodanese homology domain, similar to the human protein (Supplementary Figure S1B). The in silico results were validated by testing the ability of *zgc162544* to produce H_2_S. The *zgc162544* protein was expressed as a glutathione S-transferase fusion protein in E.coli and purified from bacterial lysates by affinity chromatography on GSH columns. SDS-PAGE proved the high purity of the isolated protein (Figure 1A). The purified GST-Zgc162544 protein’s catalytic activity was next tested using the methylene blue assay. The observed increase in H_2_S production in the presence of 3-MP provided proof for 3-Mst enzymatic activity of GST-Zgc162544 (Figure 1B) and we therefore propose that *zgc162544* is the functional orthologue of 3-MST.

### 3.2. 3-mst is Expressed Ubiquitously during Zebrafish Early Development

**Figure 2:**
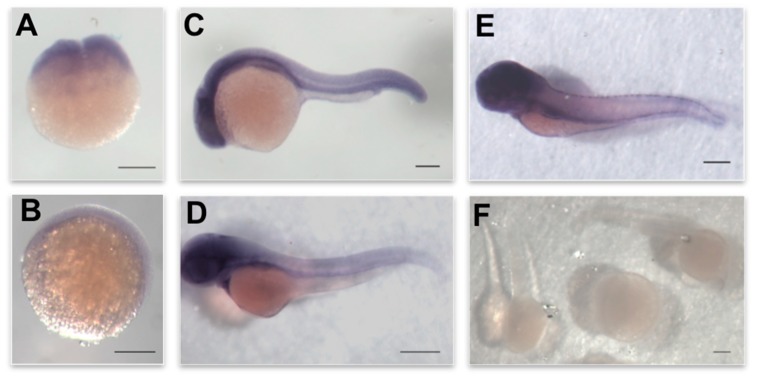
Expression pattern of *3-mst* during zebrafish development. Whole mount in situ hybridization of *3-mst* in zebrafish embryos at (**A**) 0 hpf, (**B**) 8 hpf, (**C**) 24 hpf, (**D**) 48 hpf and (**E**) 72 hpf. (**F**) Sense probe at the stages shown in (**A–E**). Scale bar 250 μΜ.

After identifying the zebrafish *3-mst* orthologue, we investigated its expression pattern during the embryos’ development. Using a specific antisense probe, we observed that *3-mst* mRNA is maternally provided, as it is widely expressed, detected already at the two-cell stage of development (Figure 2A). A broad *3-mst* expression throughout embryos was observed during the next developmental stages (Figure 2B,C). However, starting at 48 hpf, *3-mst* was predominantly expressed in the developing heart and developing zebrafish central nervous system (Figure 2D,E).

### 3.3. Generation of a 3-mst^−/−^ Zebrafish Line

**Figure 3:**
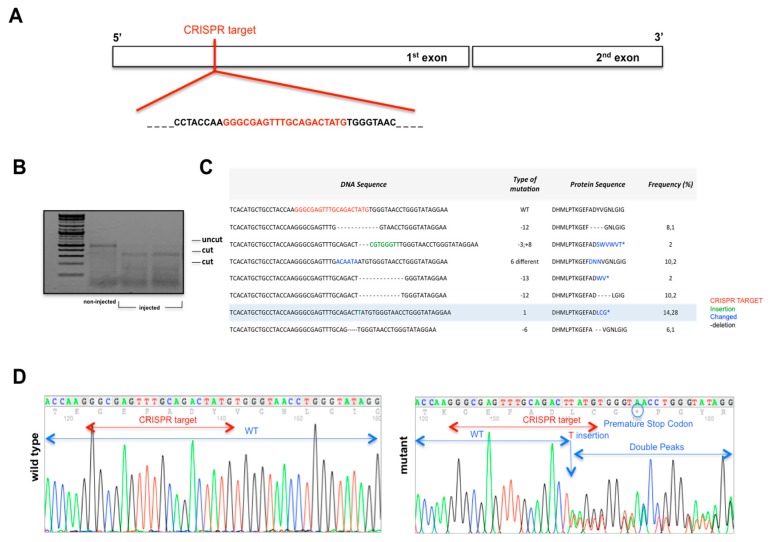
Generation of *3-mst* mutant zebrafish using the clustered, regularly interspaced, short palindromic repeats (CRISPR)-Cas9 approach. (**A**) Schematic representation of *3-mst*-CRISPR-target site. (**B**) Gel electrophoresis of T7E1 assay in a representative sampling of CRISPR-injected and non-injected controls. (**C**) Type and frequency of alleles, identified in F1 generation. (**D**) Sequencing chromatograms of wild type and the mutant allele (aa102), selected for further studies, showing the presence of a premature stop codon.

The ubiquitous expression of *3-mst* in zebrafish embryos possibly indicates the enzyme’s crucial role in development. To address this, we used the CRISPR/Cas9 system to generate a new zebrafish line with a targeted mutation in *3-mst*. Specifically, we designed a *3-mst* guide RNA that targets the first exon of the gene (Figure 3A), consequently, the predicted protein will not be functional, following the CRISPR-induced mutations. gRNA was next injected into one-cell stage embryos along with Cas9 mRNA and the targeted mutation efficiency was verified by the T7EI assay in F0 injected embryos. The appearance of T7E1 fragments indicated the positive gRNA targeting of exon 1 in the *3-mst* locus, while no T7E1 fragments were detected in non-injected control embryos (Figure 3B). After confirming the high efficiency of the CRISPR approach, F0 adults were next crossed with wild type, in order to identify founder individuals and select carriers for the appropriate alleles (Supplementary Figure S2). Genotypes of sixty F1 adult zebrafish were analyzed using sequencing analysis of the PCR-amplified *3-mst* region, spanning the *3-mst* target site. The results confirmed the generation of several *3-mst* deficient zebrafish alleles, induced by the CRISPR/Cas9 approach (Figure 3C). An allele with a single nucleotide insertion that leads to a frameshift during protein translation and an early stop codon was selected for further studies and assigned with the allele number aa102 (Figure 3D).

### 3.4. 3-mst CRISPR/Cas9 Induced Mutation in Zebrafish Leads to Reduced Levels of H_2_S

**Figure 4:**
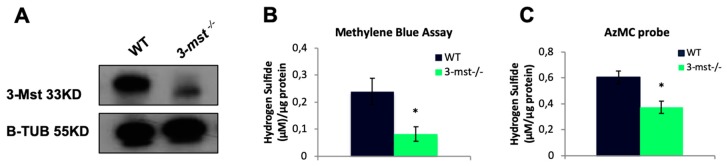
*3-mst*^−/−^ zebrafish exhibit decreased H_2_S production. (**A**) Representative Western blot shows no detectable 3-Mst protein in the *3-mst*^−/−^ larvae at 96 hpf. β-tubulin was used as a loading control. (**B**) H_2_S levels were measured by the methylene blue assay in WT and *3-mst*^−/−^ zebrafish at 96 hpf. (**C**) H_2_S levels were measured by the AzMC probe in WT and *3-mst^−/−^*zebrafish at 96 hpf. Data are presented as mean ± SEM; *n* = 5–7/genotype; * *P* < 0.05.

F1 founders, heterozygous (−/+) for the selected mutation, were next crossed to produce the F2 generation, to yield homozygous (−/−) zebrafish. F2 zebrafish with a −/− genotype were identified and mated. F3 *3-mst*^−/−^ homozygous was used for all the following experiments (Supplementary Figure S2). The efficiency of the mutation at the protein level was next confirmed using Western blot analysis. 3-Mst protein could not be detected in *3-mst*^−/−^ zebrafish embryos, indicating that the mutation leads to no protein translation (Figure 4A). Subsequently, we assessed the H_2_S levels in WT and *3-mst*^−/−^ using two different methods. In line with the Western blot results, H_2_S levels were reduced in mutant embryos, providing proof for the genetic ablation of 3-Mst function (Figure 4B,C).

### 3.5. *3-mst*^−/−^ Embryos are More Sensitive to Oxidative Stress Conditions

**Figure 5:**
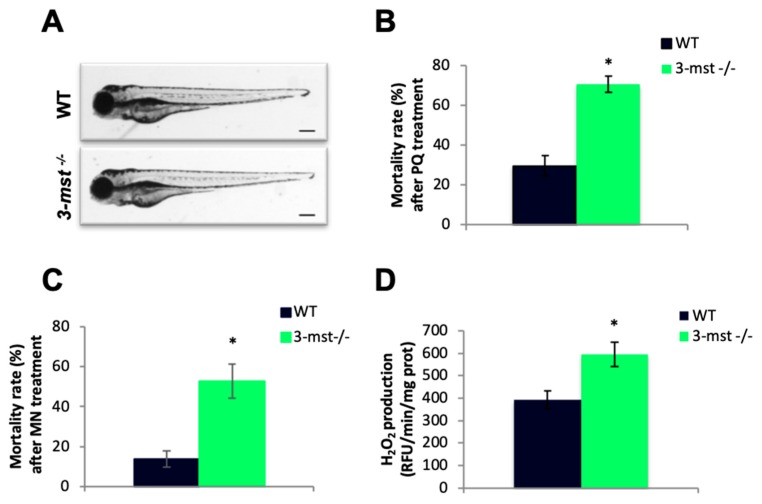
Increased H_2_O_2_ production and oxidative stress sensitivity in *3-mst*^−/−^ zebrafish. (**A**) Microscopic images show normal gross morphology of *3-mst*^−/−^ larvae at 96 hpf in comparison with WT larvae. Black scale bar: 250 μΜ. (**B**) Mortality rate of WT and *3-mst*^−/−^ larvae after PQ treatment. (**C**) Mortality rate of WT and *3-mst*^−/−^ embryos after menadione (MN) treatment (**D**) Zebrafish H_2_O_2_ levels measured by Amplex Red Fluorescence in homogenates of WT and *3-mst*^−/−^ at 96 hpf. Data are presented as mean ± SEM; *n* = 6–12/genotype; * *P* < 0.05.

*3-mst*^−/−^ zebrafish larvae survived normally and showed no growth retardation effects or obvious reproductive defects. Since we detected a maternal deposition of mRNA, we also raised maternal zygotic mutant embryos, but they also exhibited no obvious morphological deficiencies during the first developmental stages (Figure 5A). The phenotype of *3-mst*^−/−^ zebrafish was next investigated under different kinds of stress conditions. Interestingly, a higher sensitivity of mutant zebrafish to oxidative stress was noticed. The induction of oxidative stress was achieved using a sublethal concentration of paraquat (PQ), a widely used reactive oxygen species (ROS) producing agent [27]. WT and mutant zebrafish were treated with 500 μg/mL PQ at 96 hpf. After 48 h the mortality rate of larvae was determined. The deletion of *3-mst* led to increased mortality, indicating a phenotype characterized by increased sensitivity to oxidative stress (Figure 5B). This observation was next confirmed using menadione (MN), another ROS-generating agent [28]. WT and *3-mst* deficient zebrafish, aged 48 hpf, were treated with 10μΜ MN for 24 h. The increased mortality rate of mutant embryos observed following MN treatment confirmed their greater sensitivity to oxidative stress phenotype. The enhanced sensitivity of mutant embryos could be due to an altered redox balance, characterized by increased ROS levels in the absence of *3-mst*. Indeed, a significant elevation of hydrogen peroxide levels was found in *3-mst*^−/−^ zebrafish, indicating a shift towards a pro-oxidant state in mutant zebrafish (Figure 5C).

### 3.6. 3-mst Deletion Enhances Fin Regeneration

**Figure 6:**
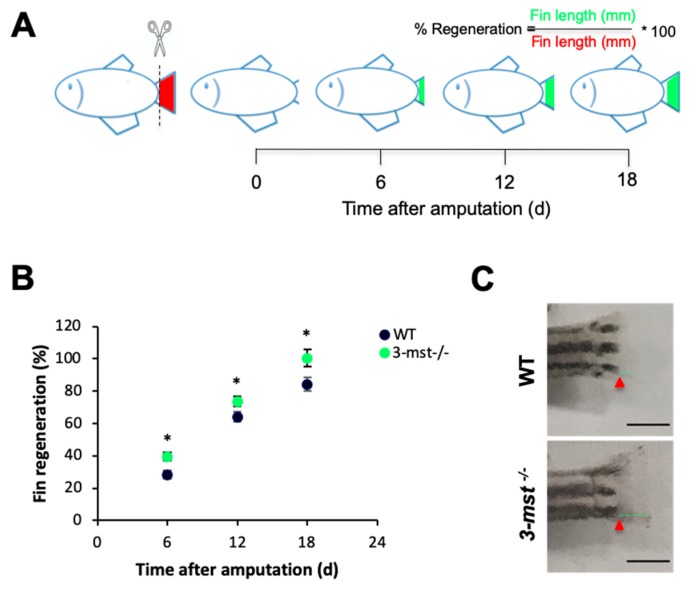
*3-mst*^−/−^ zebrafish exhibit increased caudal fin regeneration rate. (**A**) Experimental protocol. (**B**) Caudal fin regeneration rate of WT and *3-mst*^−/−^ adult zebrafish at different time points after tail amputation. (**C**) Representative fin images of WT and *3-mst*^−/−^ adult zebrafish at six days after amputation. Red arrows indicate original section plane. Green lines indicate fin length after regeneration. Black scale bar: 2 mΜ. Data are presented as mean ± SEM; *n* = 16/genotype; * *P* < 0.05.

The impact of ROS signaling on regenerative biology has been recently explored, revealing its importance for tissue regenerative growth rate [29,30,31,32]. Given the fact that *3-mst*^−/−^ zebrafish are characterized by increased levels of H_2_O_2_, the mutants’ regenerative capacity was next investigated. The caudal fins of WT and *3-mst* deficient adult zebrafish were amputated and allowed to regenerate for 18 days (Figure 6A). Measurements of regenerative fins’ length showed an increased growth rate when the enzyme is deleted (Figure 6B,C).

## 4. Discussion

In the present study, we identified the 3-MST orthologue in zebrafish and investigated its expression pattern during zebrafish early development. *3-MST* levels have been analyzed in several organs in adult rodents and have demonstrated a ubiquitous expression pattern. In mice, the cerebrum, heart, liver, kidney, testes and endocrine organs express high levels of *3-MST*, while the lungs, spleen, thymus and small intestines are characterized by a lower expression of the enzyme [33]. Cardiovascular tissues, including the heart and vessels, also contain 3-MST [34,35]. In rats, 3-MST has been successfully detected in the brain, lungs, kidneys, heart, pancreas and testis [7]. Although the expression profile of *3-MST* has been examined in various organs of adult models, its presence during development still remains poorly characterized. The brain, lung and intestines, isolated from mice, are the only tissues that have been tested for the enzyme’s expression in the early developmental stages. 3-MST was detected in all three organs during the examined stages (fetal day 12–4 weeks old), exhibiting a higher expression in brain [33]. Here, taking advantage of zebrafish characteristics, we present the expression profile of *3-mst* in whole embryo. Our data prove the ubiquitous expression of *3-mst* and confirm the predominant presence of the enzyme in the developing brain. The widespread expression of *3-mst* that we observed, is in line with the finding that its promoter shows features of a house-keeping gene [36]. Furthermore, we show for the first time that *3-mst* is highly expressed in the fetal heart, indicating a potential physiological significance of the enzyme in the cardiovascular system. Importantly, by investigating the expression of the enzyme during early development (one-cell stage embryos), we found out that *3-mst* is a maternally provided gene in zebrafish.

The early and broad expression of *3-mst* suggests an important role in development and physiology. However, only a few reports have investigated 3-MST function and signaling. We, thus, used the CRISPR/Cas9 genome-editing method to generate a *3-mst* deficient zebrafish line and morphologically characterized it during the early developmental stages. In agreement with the fact that *3-mst* knockout (KO) mice display physiologic growth, *3-mst*^−/−^ zebrafish also develop normally [8]. Furthermore, *3-mst* genetic ablation in zebrafish leads to no obvious phenotypic changes under physiological conditions. In *3-MST* KO mice, the brain is the only organ that has been examined morphologically so far, showing no gross abnormalities [8]. Next, we interrogated the importance of *3-mst* under stress conditions. Interestingly, mutants exhibited higher sensitivity (compared to WT) when they were exposed to sublethal concentrations of ROS-producing agents [27,28]. We reasoned that the enhanced sensitivity to both paraquat and menadione in *3-mst*^−/−^ might be due to basally increased endogenous ROS in the mutant line. Indeed, we detected higher H_2_O_2_ amounts in mutant larvae that would be expected to have an additive effect with PQ and MN-generated ROS. Our findings demonstrating the presence of enhanced oxidative stress in *3-mst*^−/−^ under baseline conditions are in line with the known anti-oxidant properties of 3-MST [8,37,38]. 3-MST possesses two redox-sensitive molecular switches that act as redox sensors to regulate its activity and catalyze the production of H_2_S, persulfides and polysulfides [37,39,40,41]. Persulfide formation protects protein thiols groups from irreversible oxidation [42], while hydrogen sulfide has direct and indirect antioxidant effects [43]. Although H_2_S has the potential to scavenge ROS, it is generally assumed to be a weak ROS scavenger [44]. H_2_S can modify the activity of proteins with important roles in redox homeostasis, including superoxide dismutase 1 (SOD1) and SHC-transforming protein 1 isoform (p66SHC) [42,44,45]. Moreover, H_2_S activates nuclear factor erythroid 2-related factor 2 (NRF2) by sulfhydrating the NRF2 repressor kelch-like ECH-associated protein 1 (KEAP1) [46]. The activation of NRF2 in turn, increases the expression of antioxidant genes that help restore cellular redox balance [47].

As ROS production has been linked to the initiation of the regeneration program, the ability of *3-mst*^−/−^ zebrafish to regenerate their fin after amputation was next examined [29,30,31,32]. The results from these experiments indicate that mutants are characterized by an increased regenerative potential, revealing a novel role of *3-mst* in the regulation of tissue regenerative capacity. The *3-mst* loss-of-function model we present can be used for further studies, which will contribute to the understanding of the molecular mechanism regulating tissue regeneration. Additionally, the increased ability of mutants to regenerate their caudal fin indicates that pharmacological inhibition of 3-MST could possibly be a useful strategy in regenerative medicine. Further studies, exploring the regenerative effect of enzymes deletion in other tissues and models, are necessary to support this hypothesis.

In humans, defects in 3-MST cause mercaptolactate-cysteine disulfiduria (MCDU), a congenital metabolic disorder that is associated with mental retardation [48]. Although it was reported 50 years ago, its pathogenesis has not been fully clarified yet. In mice, genetic ablation of *3-MST* was shown to result in anxiety-like behavior [8] and the *3-MST* KO mouse was proposed as a model for MCDU [8]. In recent years, the zebrafish has become an attractive model for the study of mental disorders and several behavioral assays have been developed [39,49,50]. The herein reported *3-mst* loss-of-function zebrafish could possibly become a non-mammalian model of MCDU. Future studies testing mutant zebrafish behavior would be required to validate this hypothesis.

## 5. Conclusions

Here, we present the first *3-mst^−/−^* zebrafish mutant line. It displays high sensitivity to oxidative stress, due to increased levels of reactive oxygen species and improved regenerative capacity. The study found that *3-mst* deficient embryos provide further proof of the protective role of 3-Mst in redox homeostasis and indicate a novel role of the enzyme in regeneration process. The *3-mst* mutant zebrafish represents a valuable model that can be used to clarify the function and signaling of 3-Mst and could be useful in the study of disorders associated with 3-MST deficiency.

## Supplementary Materials

The following are available online at https://www.mdpi.com/2218-273X/10/2/317/s1, Figure S1: Human 3-MST and zgc162544 protein homology. Figure S2: Schematic summarizing the method of generation and identification of mutant carrier founder zebrafish using the CRISPR/Cas system.

## Abbreviations

3-MP	3-mercaptopyruvate
*3-MST*	3-mercaptopyruvate sulfutransferase
*3-mst* ^−/−^	3-mercaptopyruvate sulfutransferase deficient
AzMc	7-azido-4-methylcoumarin
Cas9	CRISPR-associated protein-9
CBS	cystathinine-beta-synthetase
CRISPR	clustered regularly interspaced short palindromic repeats
CSE	cystathionine-gamma-lyase
GST	GSH-S-transferase
DMSO	dimethyl sulfoxide
H_2_S	hydrogen sulfide
hpf	hours post-fertilization
IPTG	Isopropyl-b-D-thiogalactopyranoside
LB	Luria–Bertani
MN	menadione
ROS	reactive oxygen species
PBS	phosphate buffered saline
PQ	paraquat
WT	Wild-type
