# Supplementary material for: Generation and Characterization of a CRISPR/Cas9—Induced 3-mst Deficient Zebrafish
Source: Biomolecules. 2020 Feb 17;10(2):317. doi: 10.3390/biom10020317 (PMC7072312; doi:10.3390/biom10020317)
Supplement: Supplementary file 1 [file biomolecules-10-00317-s001.pdf]

biomolecules

MDPI

Supplementary Materials:

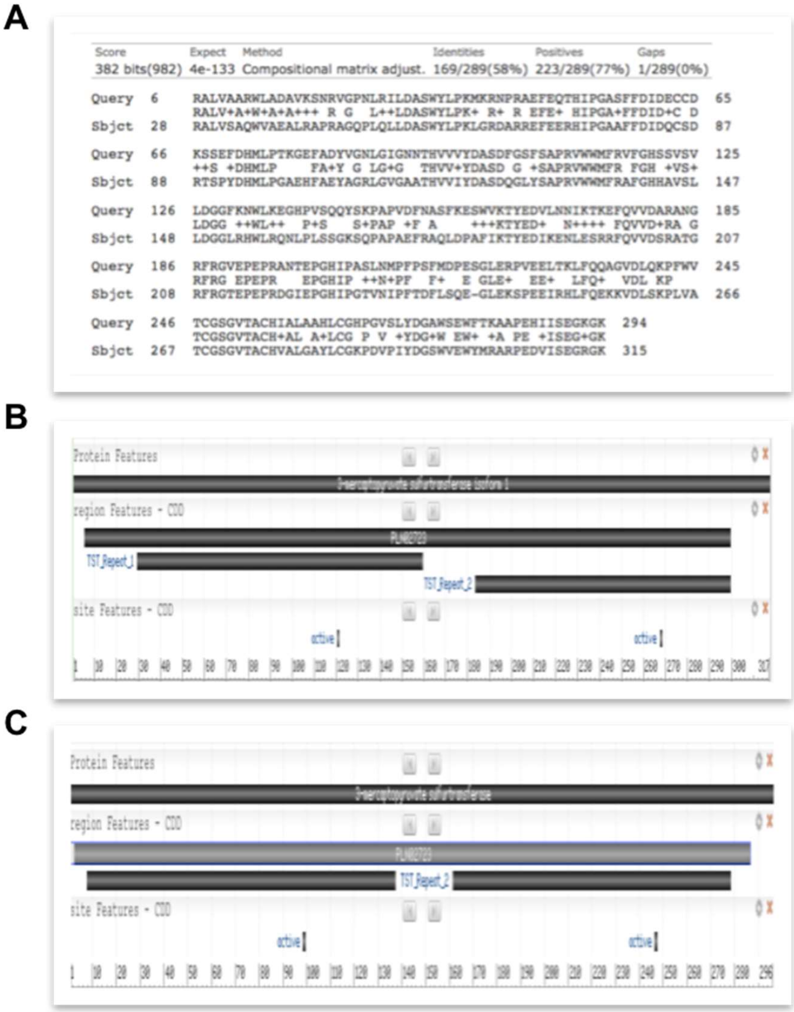

Figure S1: Human 3-MST and *zgc162544* protein homology. **(A)** Comparison at protein sequence level, according to NCBI database. **(B)** Human protein domains and **(C)** *zgc162544* protein domains, according to NCBI database. Gene IDs: 4357 (3-MST) and 796035 (*zgc162544*).

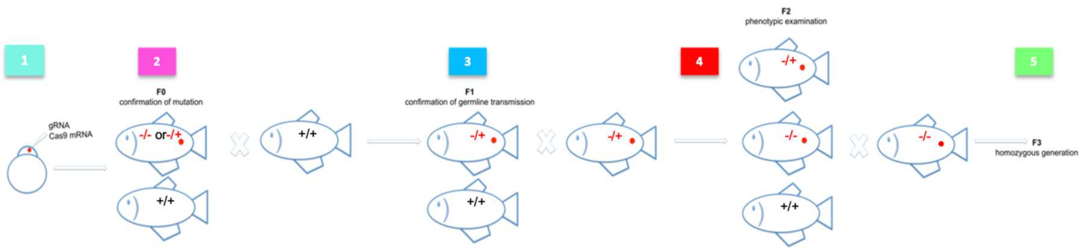

Figure S2: Schematic summarizing the method of generation and identification of mutant carrier founder zebrafish using the CRISPR/Cas system**.** **(1)** gRNAs and Cas9 mRNA injection into embryos (F0 generation), **(2)** cross of an F0 mutant (+/−or −/−) with a wild type (+/+) (F2 generation), **(3)** confirmation of germline transmission in F1 zebrafish generation, **(4)** mating of F1 −/+ founders with same type of mutation to identify −/− mutants (F2 generation) and **(5)** in cross of −/− founders to produce the F3 homozygous (and maternal zygotic mutants) for the phenotype characterization.
